# A Systematic Review on the Impact of Pregnancy on Renal Graft Function

**DOI:** 10.3390/jcm14145022

**Published:** 2025-07-16

**Authors:** Beatriz Banuelos Marco, Muhammet Irfan Donmez, Batuhan Erkul, Hakan Bahadir Haberal, Alessio Pecoraro, Thomas Prudhomme, Riccardo Campi, Alberto Piana, Alicia Lopez-Abad, Romain Boissier, Albert Breda, Angelo Territo

**Affiliations:** 1 Department of Urology, Renal Transplant Division, University Hospital Clínico San Carlos, 28040 Madrid, Spain; 2 Department of Urology, Istanbul Faculty of Medicine, Division of Pediatric Urology, Istanbul University, 34093 Istanbul, Turkey; 3 Department of Urology, Ankara Ataturk Sanatorium Training and Research Hospital, Ministry of Health, University of Health Sciences, 06290 Ankara, Turkey; 4 Unit of Urological Robotic Surgery and Renal Transplantation, Careggi Hospital, University of Florence, 50134 Florence, Italy; 5 Department of Urology and Kidney Transplantation, Rangueil University Hospital, 31400 Toulouse, France; 6 Department of Experimental and Clinical Medicine, University of Florence, 50134 Florence, Italy; 7 Department of Urology, San Luigi Gonzaga Hospital, University of Turin, 10043 Turin, Italy; 8 Department of Urology, Virges de La Arrixaça University Hospital, 30120 Murcia, Spain; 9 Department of Urology and Renal Transplantation, La Conception University Hospital, 13005 Marseille, France; 10 Uro-Oncology and Kidney Transplant Unit, Department of Urology at “Fundació Puigvert” Hospital, Autonoma University of Barcelona, 08193 Barcelona, Spain

**Keywords:** kidney transplant, renal transplant, pregnancy complications, graft function, urological complications, adverse events, pregnancy outcome, maternal outcome, fetal outcome, birth outcome

## Abstract

**Background/Objectives:** Renal transplantation (RT) represents the optimal treatment for end-stage renal disease (ESRD), offering improved quality of life and restored fertility in women post-transplant. While post-transplant pregnancies are possible, they can lead to complications including pre-eclampsia, graft dysfunction, and other adverse outcomes. This study evaluates existing literature to assess pregnancy’s impact on kidney transplantation outcomes, specifically long-term graft function and survival. **Methods:** We conducted a systematic review of English-language literature from January 2000 to September 2023 across multiple databases, following PRISMA guidelines. We established inclusion criteria focusing on graft function and adverse events. Two independent reviewers performed data extraction, and we assessed risk of bias using the ROBINS-I tool. **Results:** From 4917 articles, we included 26 studies encompassing 1202 pregnancies in 902 kidney transplant recipients. Mean maternal age was 30.8 years, with an average interval of 52 months between transplant and pregnancy. Pre-pregnancy hypertension occurred in 54.2% of cases, and pre-eclampsia developed in 25.7%. The live birth rate reached 70.5%, while miscarriage, stillbirth, and neonatal death rates were 11.3%, 2.7%, and 2.5%, respectively. We noticed graft dysfunction during pregnancy in 20.2% of cases. Though kidney function often deteriorated temporarily, most patients recovered post-delivery. **Discussion:** Post-transplant pregnancies remain viable but high-risk, with elevated rates of obstetric complications. Our findings highlight the need for standardized data collection and reporting to better understand and manage pregnancy’s impact on graft outcomes. **Conclusions:** With appropriate management, pregnancy in kidney transplant recipients is feasible, though it carries elevated risks of obstetric complications. We recommend further multicenter studies with standardized data collection to improve understanding and outcomes.

## 1. Introduction

Kidney transplantation (KT) offers the best treatment option for end-stage renal disease (ESRD), enhancing quality of life and restoring fertility in women within 6 months post-transplantation [1]. While pregnancy rarely occurs in women with ESRD on dialysis (0.9–7% conception rate) [2], successful pregnancies become possible after KT, enabling many couples to achieve parenthood.

Pregnancy presents unique challenges for transplant recipients. The gestational period brings significant changes in body metabolism, drug processing, and immune function. Transplant recipients face increased risks of complications, including pre-eclampsia, graft dysfunction, and in rare cases, recipient mortality [3]. Even after successful pregnancy completion, patients may experience disruptions in renal graft function that could compromise long-term outcomes. The relationship between pregnancy and renal transplantation has undergone extensive investigation.

This study aims to synthesize the available literature and provide insights into pregnancy’s potential impact on kidney transplantation outcomes, particularly regarding long-term graft function and overall survival.

## 2. Materials and Methods

### 2.1. Data Sources and Searches

**Figure 1:**
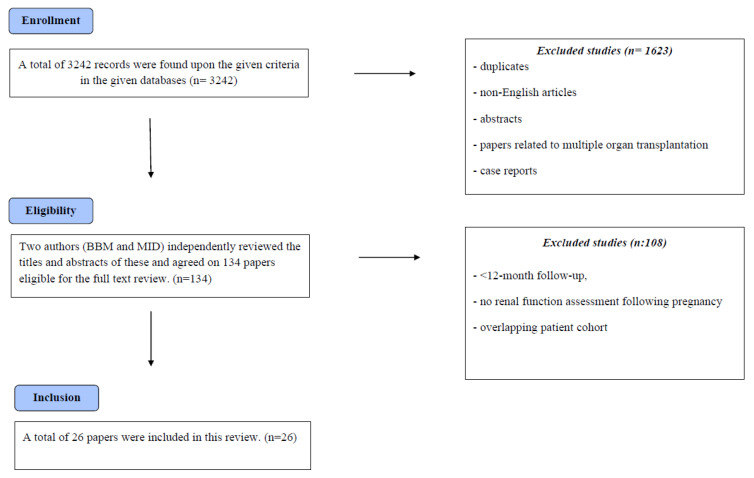
Flowchart Study Selection.

We conducted a systematic review following PRISMA guidelines (PROSPERO ID: CRD42023456893) of English-language literature published between January 2000 and September 2023. Our search covered PubMed/MEDLINE, Elsevier EMBASE, Scopus, BIOSIS Previews, ISI Science Citation Index Expanded, and the Cochrane Central Register of Controlled Trials (CENTRAL). We also reviewed abstracts from annual American Transplant Congresses examining pregnancy incidence and outcomes in kidney transplant recipients (Figure 1). A health sciences librarian (E.K.) developed database-specific search strategies combining subject headings (MeSH or Emtree) with keywords. Key search terms included pregnancy complications, pregnancy outcome, maternal outcome, fetal outcome, birth outcome, kidney transplant, and renal transplant.

### 2.2. Study Selection

Two independent reviewers (B.B. and M.I.D.) screened titles and abstracts of identified citations to determine eligibility for full-text review. A third author (A.T.) resolved any discrepancies. We excluded duplicate studies, non-English literature, abstracts without full text, papers on multiple organ transplantation, and case reports. Additional exclusion criteria included studies with less than 12 months of follow-up, those lacking renal function assessment after pregnancy, and those with overlapping patient cohorts. Two team members (B.B. and M.I.D.) established the inclusion/exclusion criteria, which all EAU YAU KT WG member co-authors reviewed to ensure focus on graft function and adverse events.

### 2.3. Data Extraction, Quality Assessment

Two team members (B.B. and B.E.) independently extracted data using standardized forms as previously described [4]. B.B. rechecked all data elements for accuracy. For multiple publications with over 25% patient population overlap, we included the study with the larger number of pregnancy events and the most complete details. An arbitrator (M.I.D.) and primary investigator (B.B.) resolved any disagreements in data extraction and quality assessment.

We extracted the following data when available:

- Country and data collection period.
- Number of KT recipients and pregnancies.
- Mean maternal age and interval between KT and pregnancy.
- Pregnancy outcomes (live births, miscarriages, induced abortions, stillbirths, ectopic pregnancies).
- Maternal outcomes (pre-eclampsia, pregnancy-induced hypertension, gestational diabetes mellitus, cesarean sections).
- Fetal outcomes (pre-term births, gestational age, birth weight, neonatal deaths).
- Graft outcomes (acute rejection during pregnancy, post-pregnancy graft failure, serum creatinine before and after pregnancy).

For consistency, we used pregnancy numbers as denominators for pregnancy outcomes and patient numbers for graft outcomes. We defined pre-term birth as delivery before 37 weeks’ gestation.

We evaluated study risk of bias using the Risk of Bias in Non-Randomized Studies of Interventions (ROBINS-I) tool [5]. This Cochrane-Reviews-recommended tool helped categorize overall risk of bias as “low,” “unclear,” or “high” (Table 1). Study heterogeneity precluded meta-analysis.

The description of the study methodology is provided as Supplementary Material.

## 3. Results

Our search identified 4917 citations, leading to 134 full-text article reviews. We selected 26 studies for final inclusion (Table 1) [6,7,8,9,10,11,12,13,14,15,16,17,18,19,20,21,22,23,24,25,26,27,28,29,30,31], comprising 1202 pregnancies among 902 kidney transplant recipients. Recipients averaged 25.8 ± 4.7 years at transplantation and 30.8 ± 4.5 years at pregnancy. The mean transplant-to-pregnancy interval was 52 months (range 24–79). Donor sources included 413 (54%) deceased and 364 (46%) living donors across 22 studies reporting this information.

Seven studies documented transplant sequence: 352 first transplants and 64 s transplants among 416 patients. Only two studies reported previous acute rejections (2/10 and 5/46) and previous abortions (2/10 and 1/46).

Nineteen studies detailed ESRD etiology for 594 patients (Table 2). Only three studies specifically reported urological comorbidities, affecting 16/85 patients (18%).

### 3.1. Immunosuppression

Most studies reported pre-conception immunosuppression changes, primarily switching from mycophenolate to azathioprine. Among studies providing absolute numbers, 260/298 patients (87%) underwent immunosuppression regimen changes. Double immunosuppression was used in 283 patients (46%), while triple immunosuppression was employed in 340 patients (54%). Table 3 details immunosuppression ratios across included studies.

### 3.2. Pregnancy Outcomes

Twenty studies reported pre-pregnancy comorbidities, though documentation varied. Hypertension affected 393/724 patients (54.2%), while diabetes mellitus occurred in 36/341 (10.5%). Twenty-two studies defined successful pregnancy as reaching >12 weeks’ gestation, with 646/786 pregnancies (82%) meeting this criterion. Maternal adverse events showed inconsistent reporting across studies. Pre-eclampsia occurred in 210/815 patients (25.7%) across 21 studies.

Seventeen studies documented 669 pregnancy outcomes: 472 live births (70.5%), 86 voluntary abortions (12.8%), 76 spontaneous abortions/miscarriages (11.3%), 18 stillbirths (2.7%), and 17 neonatal deaths (2.5%). Gestational diabetes affected 12/157 patients (7.6%) in five reporting studies.

Acute kidney injury (AKI) occurred in 18/517 cases (34.5%) across three studies. Aivazoglou et al. [26] found that 10 out of 30 patients developed AKI related to previous pre-eclampsia. Seventeen studies reported hypertension onset in 127/564 patients (22.3%), hypertension worsening in 107 patients out of 564 (18.8%), and hyperfiltration (GFR > 125 mL/min) in 112 patients out of 492 (22.7%). Three studies documented eclampsia, with rates varying from 1 out of 10 to 2 out to 22 and 1 out of 72 patients. One maternal death occurred due to eclampsia. Twelve studies reported pregnancy-related anemia (62/223, 27.8%).

### 3.3. Delivery and Related Adverse Events

Delivery methods included 193/517 (37.3%) vaginal deliveries and 324/517 (62.7%) cesarean sections. Three studies indicated that obstetric indications prompted 38/61 (62.2%) cesarean sections. Delivery complications varied across studies: hemorrhage affected 19/143 (13.2%), induced delivery occurred in 13/112 (11.6%), major placenta previa in 3/33 (9%), and premature membrane rupture in 2/16 (12.5%).

### 3.4. Kidney Transplant Adverse Events

Twenty-three studies documented graft dysfunction during pregnancy, defined as a >20% serum creatinine increase from the pre-conception baseline, affecting 169/838 patients. Overall kidney transplant adverse events occurred in 258/839 patients across 24 studies (Table 4). Urinary tract infections and acute pyelonephritis frequently complicated pregnancies, affecting 47/265 and 14/265 patients, respectively.

### 3.5. Renal Function

Pre-pregnancy renal function measurements showed mean creatinine levels of 1.16 mg/dL (range 0.88–1.4) in 20 studies. Eight studies reported mean pre-pregnancy eGFR of 57.7 mL/min/1.73 m^2^ (range 47.9–64). At delivery, eleven studies showed mean creatinine of 1.17 mg/dL (range 0.9–1.27) and mean eGFR of 51.7 mL/min/1.73 m^2^ (range 46–57.5).

Three months post-delivery, seven studies reported mean creatinine of 1.28 mg/dL (range 0.96–1.6). At twelve months, fourteen studies showed mean creatinine of 1.28 mg/dL (range 0.98–1.51) and three studies reported mean eGFR of 55.7 mL/min/1.73 m^2^ (range 49–59.2).

Mean follow-up duration reached 81 months, excluding two studies lacking exact timing but confirming >12-month post-delivery follow-up. Final mean creatinine levels across 22 studies reached 1.28 mg/dL (range 1.03–1.79), while three studies reported mean eGFR of 55.1 mL/min/1.73 m^2^ (range 47–59.2).

Graft loss occurred in 8/810 patients. Post-pregnancy acute rejection affected 15/242 patients (6%) across ten studies, while chronic rejection occurred in 19/197 patients (9.7%) across seven studies. Graft survival rates were 90% at 5 years (six studies), 77.1% at 10 years (five studies), and 68.9% at 15 years (four studies). Eight studies reported 155/166 transplanted women maintained functioning grafts at follow-up conclusion. Seven studies documented 47 maternal deaths among 472 women (10%) during follow-up.

## 4. Discussion

This systematic review, encompassing 26 studies from 20 countries and representing 1202 pregnancies in 902 kidney transplant recipients, confirms that successful pregnancies are achievable post-transplantation. The live birth rate exceeded the general US population (70.5% vs. 66.7%), while miscarriage rates remained lower (15.4% vs. 17.1%) [32,33] Though viable, post-transplant pregnancies carry elevated obstetric risks. Compared to US averages, these pregnancies showed higher rates of gestational diabetes (10.5% vs. 8.3%), pre-eclampsia (25.7% vs. 5–7%), cesarean delivery (62.7% vs. 32.1%), and pre-term birth (35.5% vs. 10.4%). Neonatal mortality exceeded European population rates (2.5% vs. 0.2%) [34]. Recipients typically delivered late pre-term (34.8 weeks) with low birth weight (2457.5 g) infants.

Renal function typically deteriorates during pregnancy but recovers within the first post-delivery year, particularly in patients with lower initial GFR values. This suggests pregnancy may not threaten graft survival as previously feared. However, inconsistent reporting of creatinine values and eGFR complicates data analysis and comparison [35]. Graft survival rates reached 90% at 5 years (six studies), 77.1% at 10 years (five studies), and 68.9% at 15 years (four studies). These outcomes align with US Scientific Registry of Transplant Recipients (SRTR) data, showing 78.1% 5-year survival for 2014–2017 and 76.3% and 51.6% at 5 and 10 years, respectively, for 2010–2013 [36].

Eight studies reported functioning graft status at follow-up conclusion, with 155/166 transplanted women maintaining viable grafts. One notable study investigated acute kidney injury (AKI) in patients with pre-transplant pre-eclampsia, finding that 10 out of 30 (33.3%) patients experienced AKI related to previous pre-eclampsia [26]. This finding provides crucial insight into pregnancy’s effects on renal allografts, though most studies lack such detailed analysis.

Recent decades have seen improved graft survival rates due to advances in immunosuppressive therapy. These improvements enable transplant recipients to reach adulthood when transplanted in childhood or achieve childbearing age. However, some patients require second or third transplantations, potentially increasing immunological burden, particularly when considering pregnancy. Our review found only seven studies documenting transplant sequence (352 first and 64 s transplants among 416 patients). Just two studies reported previous acute rejections (2/10 and 5/46) and previous abortions (2/10 and 1/46). This information proves crucial for understanding hyperimmunization risks and second transplant failure rates, particularly relevant for women of childbearing age. Additionally, no studies examined intervals shorter than one year post-transplant, leaving questions about early post-transplant pregnancy risks and their physiological mechanisms unanswered.

Our findings align with data from large voluntary registries in the US and United Kingdom (UK), which report live birth rates of 71–79% and miscarriage rates of 12–24% among KT recipients [32]. These consistencies across our meta-analysis suggest the National Transplant Pregnancy Registry (NTPR) and UK registry serve as reliable clinical benchmarks. We strongly advocate continued post-transplantation pregnancy data reporting.

Several limitations merit consideration. Despite efforts to exclude duplicate cohorts, registry analyses may contain overlapping patient data. Geographic variations in peri-natal care, nutrition, socioeconomic status, and healthcare infrastructure potentially confound results. Diagnostic criteria for conditions like pre-eclampsia and hypertension vary between institutions, complicating classification. Some studies inadequately define successful pregnancy or fail to distinguish between therapeutic and elective abortions. Furthermore, the predominance of retrospective studies introduces potential selection and reporting biases. Live birth outcomes show encouraging international trends, suggesting possible graft preservation among KT recipients. However, practitioners must remain vigilant regarding obstetric complications. Van Buren’s study highlighted concerning rates of graft dysfunction (90/197 patients) and pre-eclampsia (33 patients with pre-existing hypertension) [23] Delivery complications, including cesarean sections and pre-term births, can significantly impact both maternal and fetal health. While our analysis documented one eclampsia-related maternal death, overall maternal mortality reporting proved inconsistent. For context, European maternal mortality in 2020 was 6/100,000 [37]. These elevated complication rates reinforce the high-risk classification of post-KT pregnancies.

The relative scarcity of urological complication reporting, despite being a primary review focus, potentially obscures important graft-affecting processes during and after pregnancy. This gap suggests the need for more comprehensive documentation of urological outcomes in future studies.

## 5. Conclusions

While pregnancy after kidney transplantation appears safe for mother, child, and graft, outcome reporting remains inconsistent. We recommend standardizing data collection for future multicenter studies, with particular attention to urological complications. The EAU YAU KT WG could provide an ideal framework for developing comparable worldwide data collection and analysis protocols.

## Figures and Tables

**Table 1:** Risk of Bias Summary: Green: Low risk of bias, Yellow: Unclear risk of bias, Red: High risk of bias.

Article/Bias	Due to Confounding	Selection of Participants	Classification of Interventions	Deviations from Intended Intervention	Due to Missing Data	Measurement of Outcomes	Selection of the Reported Result
STAVART et al. [6]	+	-	+	+	-	+	NI
AREIA et al. [7]	-	-	+	-	NI	+	+
CELIK et al. [8]	-	-	+	NI	NI	+	+
DI LORETO et al. [9]	-	-	-	NI	NI	-	+
DIAZ GOMEZ et al. [10]	-	-	-	+	NI	+	+
DEBSKA et al. [11]	+	-	+	+	NI	+	-
FISCHER et al. [12]	+	+	+	NI	+	+	NI
FARR et al. [13]	-	-	-	+	-	+	+
GORGULU et al. [14]	-	-	+	NI	NI	+	+
HOOI et al. [15]	+	+	+	+	-	+	+
KIM et al. [16]	+	+	+	NI	+	+	NI
ROCHA et al. [17]	+	-	-	+	NI	+	NI
LITTLE et al. [18]	+	-	-	NI	NI	-	NI
KOVACJ et al. [19]	+	-	-	+	+	+	NI
SCHWARZ et al. [20]	+	+	+	+	-	+	NI
OZBAN et al. [21]	+	-	+	+	NI	-	NI
KAATZ et al. [22]	+	-	+	+	+	+	NI
van BUREN et al. [23]	+	-	-	+	-	+	NI
RAHAMIMOV et al. [24]	+	+	+	+	+	+	+
ABE et al. [25]	-	+	+	+	+	+	+
AIVAZOGLOU et al. [26]	+	+	+	NI	+	+	NI
GALDO et al. [27]	-	+	-	+	NI	+	+
GUTIEREZ et al. [28]	-	-	-	+	NI	-	NI
KWEK et al. [29]	+	+	+	NI	+	+	-
THOMPSON et al. [30]	+	+	+	NI	+	+	+
KATTAH et al. [31]	+	-	+	+	+	+	+

**Table 2:** Overview of end-stage renal disease etiologies of the patients involved in the cohort.

Condition	n
Glomerulonephritis	155
Multicystic kidney disorders	67
IgA nephropathy	50
Interstitial nephritis	43
CAKUT (VUR, hypodysplasia, etc.)	38
Diabetic nephropathy	19
Mediterranean fever	18
Autoimmune disease	17
Polycystic kidney disease	12
Renovascular problems	10
Nephronophthisis	4
Chronic pyelonephritis	3
Other reasons	66
Unknown	64
**Total**	**594**

**Table 3:** Overview of immunosuppressive agents used during pregnancy.

Immunosuppression	n	%
Steroids	197	14%
Methylprednisolone	499	35.7%
Cyclosporine	312	22.3%
Calcineurin inhibitor (unspecified)	59	4.2%
Tacrolimus	118	8.4%

**Table 4:** Pregnancy-related outcomes in the studies included in the systematic review. N: number, GD: graft dysfunction, GL: graft loss, KT: kidney transplantation, UTI: urinary tract infection, PNA: acute pyelonephritis.

Study	Year Published	Period	Country	Recipients	Pregnancies	N	GD n/%	GL n/%	Acute Rejection n/%	KT Toxemia n/%	Proteinuria n/%	KT Obstruction n/%	UTI n/%	PNA n/%
Kovac et al. [19]	2021	1970–2016	Slovenia	18	22	18	NA	NA	0 (0)	NA	9 (50)	NA	NA	NA
Schwarz et al. [20]	2022	1972–2019	Germany	67	92	67	1 (1.5)	1 (1.5)	0 (0)	NA	20 (29)	9 (13.4)	0 (0)	0 (0)
Ozban et al. [21]	2019	2006–2018	Turkey	5	6	5	NA	NA	NA	NA	2 (40)	NA	NA	NA
Kaatz et al. [22]	2023	2003–2016	Germany	40	43	40	0 (0)	0 (0)	NA	NA	20 (50)	0 (0)	0 (0)	0 (0)
Kattah et al. [31]	2022	1996–2014	USA	37	46	37	1 (2.7)	0 (0)	1 (2.7)	NA	0 (0)	NA	6 (16)	0 (0)
van Buren et al. [23]	2022	1971–2002	Netherlands	197	295	197	90 (45.6)	1 (0.5)	NA	NA	NA	NA	NA	NA
Rahaminov et al. [24]	2006	1983–1998	Israel/Germany	39	69	39	0 (0)	0 (0)	0 (0)	0 (0)	2 (5)	0 (0)	0 (0)	0 (0)
Abe et al. [25]	2008	1977–2002	Japan	20	21	20	8 (40)	0 (0)	NA	8 (40)	NA	NA	NA	NA
Aivazoglou et al. [26]	2011	2006–2010	Brazil	30	34	30	15 (50)	1 (3.33)	2 (6.66)	0 (0)	1 (3.33)	1 (3.33)	1 (3.33)	0 (0)
Galdo et al. [27]	2005	1982–2002	Chile	30	37	30	10 (33.3)	0 (0)	5 (16.65)	0 (0)	0 (0)	0 (0)	5 (16.65)	0 (0)
Gutierrez et al. [28]	2005	1976–2004	Spain	35	43	35	2 (5.7)	0 (0)	0 (0)	0 (0)	5 (14.3)	0 (0)	0 (0)	0 (0)
Kwek et al. [29]	2015	2001–2012	Singapore	9	10	9	1 (1.1)	0 (0)	0 (0)	NA	3 (3.3)	NA	NA	NA
Thompson et al. [30]	2003	1976–2001	UK	24	48	24	5 (20.8)	0 (0)	0 (0)	0 (0)	0 (0)	0 (0)	7 (29.16)	2 (8.3)
Stavart et al. [31]	2023	2000–2021	Switzerland	10	14	10	6 (60)	0 (0)	9 (90)	0 (0)	NA	NA	6 (60)	6 (60)
Areia et al. [7]	2009	1989–2007	Portugal	28	34	28	2 (7.14)	0 (0)	1 (3.5)	NA	1 (3.5)	NA	NA	NA
Celik et al. [8]	2011	1998–2008	Turkey	24	31	24	3 (12.5)	0 (0)	0 (0)	5 (20.8)	0 (0)	NA	NA	NA
Di Loreto et al. [9]	2010	1997–2009	Italy	12	13	12	NA	0 (0)	0 (0)	1 (8)	1 (8)	NA	1 (8)	0 (0)
Diaz Gomez et al. [10]	2008		Spain	10	10	10	NA	0 (0)	2 (20)	2 (20)	0 (0)	NA	NA	NA
Debska–Slizien et al. [11]	2014	1980–2012	Poland	17	22	17	0 (0)	0 (0)	0 (0)	0 (0)	1 (5.9)	NA	NA	NA
Fischer et al. [12]	2005		Germany	81	81	81	NA	5 (6)	NA	0 (0)	NA	NA	NA	NA
Farr et al. [13]	2014		Austria	12	10	12	3 (25)	NA	NA	NA	NA	NA	NA	NA
Gorgulu et al. [14]	2010	1983–2008	Turkey	19	22	19	2 (10.5)	NA	NA	NA	2 (10.5)	NA	4 (21)	0 (0)
Hooi et al. [15]	2003	1975–2001	Malaysia	46	72	46	15 (32.6)	0 (0)	0 (0)	NA	11 (23.9)	NA	9 (19.5)	0 (0)
Kim et al. [16]	2008	1991–2005	Korea	48	74	48	0 (0)	0 (0)	1 (2)	NA	1 (2)	NA	2 (4)	0 (0)
Rocha et al. [17]	2013	1983–2009	Portugal	24	24	24	0 (0)	0 (0)	0 (0)	NA	3 (12)	NA	6 (24)	6 (24)
Little et al. [18]	2000	1985–1998	Ireland	19	29	19	5 (26.3)	0 (0)	1 (34.5)	NA	0 (0)	NA	NA	NA
**Total**				902	1202	902	169/838 (20)	8/810 (9.8)	22/460 (4.8)	16/220 (7.2)	78/508 (15.35)	10/265 (3.8)	47/265 (17.8)	14/265 (5)

## Data Availability

A systematic review of the English-language literature according to the PRISMA guidelines (PROSPERO ID: CRD42023456893) was conducted for the published literature between January 2000 and September 2023, using PubMed/MEDLINE, Elsevier EMBASE, Scopus, BIOSIS Previews, ISI Science Citation Index Expanded, and the Cochrane Central Register of Controlled Trials (CENTRAL) (from their earliest date of inception through 8/31/2017) and abstracts from the annual American Transplant Congresses exploring incidence and outcomes of pregnancy in women with kidney transplant (Figure 1).

## Supplementary Materials

The following supporting information can be downloaded at: https://www.mdpi.com/article/10.3390/jcm14145022/s1.

## Abbreviations

The following abbreviations are used in this manuscript:

European Association of Urology Young Academic Urologists Kidney Transplant Working Group (EAU YAU KT WG), Kidney Transplantation (KT), End-Stage Renal Disease (ESRD), Estimated Glomerular Filtrate Rate (eGFR), Hypertension (HT), Diabetes Mellitus (DM), Acute Kidney Injury (AKI), Graft Dysfunction (GD).
