# Supplementary material for: A Systematic Review on the Impact of Pregnancy on Renal Graft Function
Source: J Clin Med. 2025 Jul 16;14(14):5022. doi: 10.3390/jcm14145022 (PMC12295975; doi:10.3390/jcm14145022)
Supplement: Supplementary file 1 [file jcm-14-05022-s001.zip › jcm-3665360-supplementary.pdf]

Study	Methodological Explanation
Kovac et al. (2021)	Retrospective study on pregnancy outcomes in kidney transplant recipients by reviewing medical records of post-transplant pregnancies managed at a single center.
Schwarz et al. (2022)	Multicenter retrospective cohort study using data from a national registry to assess long-term pregnancy outcomes after kidney transplantation.
Ozban et al. (2019)	Single-center retrospective study analyzing recent outcomes of pregnancies in kidney transplant recipients.
Kaatz et al. (2023)	Retrospective analysis of pregnancy outcomes in a transplant population followed over 13 years.
Kattah et al. (2022)	Retrospective study based on patient chart review to determine the maternal and graft outcomes among transplant recipients.
van Buren et al. (2022)	Retrospective cohort study covering three decades of pregnancy outcomes in a Dutch kidney transplant population.
Rahaminov et al. (2006)	Retrospective study analyzing maternal and fetal complications post-kidney transplant.
Abe et al. (2008)	Retrospective observational study examining pregnancy outcomes in kidney transplant recipients.
Aivazoglou et al. (2011)	Retrospective cohort study looking into both maternal and fetal complications post-transplant.
Galdo et al. (2005)	Retrospective review of medical records in patients with kidney transplants who became pregnant.
Gutierrez et al. (2005)	Retrospective single-center study focusing on maternal outcomes after transplantation.
Kwek et al. (2015)	Retrospective chart-based study analyzing pregnancy in renal transplant recipients.
Thompson et al. (2003)	Retrospective study covering 25 years of post-transplant pregnancies.

Stavart et al. (2023)	Retrospective case series on pregnancy management and complications post-transplant.
Areia et al. (2009)	Retrospective study focusing on clinical outcomes during and after pregnancy in transplant recipients.
Celik et al. (2011)	Retrospective case series examining maternal and graft outcomes.
Di Loreto et al. (2008)	Retrospective study analyzing a cohort of post-transplant pregnancies.
Diaz Gomez et al. (2008)	Small retrospective study investigating complications in pregnant transplant recipients.
Debska–Slizien et al. (2014)	Retrospective cohort focused on pregnancy and graft function.
Fischer et al. (2005)	Large retrospective analysis assessing maternal and neonatal outcomes.
Farr et al. (2014)	Retrospective case series evaluating the success of pregnancies post-transplant.
Gorgulu et al. (2010)	Retrospective observational study with detailed maternal and fetal monitoring.
Hooi et al. (2003)	Retrospective cohort study over two decades of follow-up.
Kim et al. (2008)	Retrospective study assessing graft survival and obstetric outcomes.
Rocha et al. (2013)	Retrospective study reviewing charts of pregnant kidney transplant patients.
Little et al. (2000)	Retrospective observational study on post-transplant pregnancy outcomes.
